# Evaluating voter perceptions of political party similarity: A mixed-method study of party positions in Taiwan

**DOI:** 10.1371/journal.pone.0335465

**Published:** 2025-10-29

**Authors:** Shun-Chuan Chang

**Affiliations:** Holistic Education Center, Mackay Medical University, New Taipei City, Taiwan; National Cheng Kung University, TAIWAN

## Abstract

This study examines voter perceptions of political party similarity using data from a validated online survey conducted in Taiwan. It primarily collects qualitative data through open-ended questions, complemented by Multiple Correspondence Analysis (MCA) and feature matching techniques. The findings reveal that party competition in Taiwan is multidimensional, extending beyond traditional blue-green and unification-independence divides. Notably, local Taiwanese issues and social concerns have become increasingly prominent among emerging third parties. Feature matching results show that 22.53% of respondents clearly distinguish the Taiwan People’s Party (TPP), while 11.42% identify the New Power Party (NPP), differentiating it from the pan-green camp as part of the emerging third force. Taiwan’s unique political context, shaped by democratization, cross-strait tensions, and the rise of influential third parties, provides valuable insights for comparative politics. The study offers an analytical framework for understanding party system evolution in emerging democracies and deepens our grasp of how identity politics and diverse political engagement transform political competition. This framework enables scholars to systematically capture complex voter perceptions in multi-party systems and facilitates comparative analysis across political environments marked by identity-based polarization and increasing party plurality.

## Introduction

In the literature on spatial models of politics, one-dimensional frameworks—such as the liberal-conservative or left-right spectrum—have traditionally served as the primary lens for analyzing political ideologies and party dynamics. This ideological divide has played a central role in shaping party systems in Western democracies, with the right emphasizing individual initiative and accepting social inequalities, and the left advocating for expanded government intervention to reduce these inequalities [1]. While these models effectively explain party positions, including those at the political extremes, they may not fully capture the complexity of political ideologies and their influence on voter perceptions [2–5].

This study addresses a critical gap in spatial models of politics by examining voter perceptions of political party similarity, with a specific focus on Taiwan’s evolving party system. Taiwan’s unique political environment—shaped by democratization, cross-strait tensions, and the rise of influential third parties challenging traditional two-party dominance—provides a valuable context for exploring how voters cognitively organize parties. By moving beyond conventional left-right ideology, this research intends to reveal more complex dimensions of party competition and voter alignment, shedding light on Taiwan’s dynamic political landscape and contributing to democratic theory.

The research employs a voter-centric, exploratory approach utilizing an online survey where respondents articulate how they mentally group parties and explain their reasoning. Focusing on direct voter perceptions rather than predefined ideological scales, this approach offers a richer understanding of how parties are perceived. The study applies qualitative coding alongside quantitative analysis to interpret survey responses. This inquiry is crucial for advancing statistical models of political competition and insights into voter behavior. Ultimately, the research aims to analyze how voters construct perceptions of party similarity and how these perceptions influence party competition dynamics and voter alignment.

### Literature review

Rooted in rational choice theory as introduced by Anthony Downs [6], spatial models propose that political parties strategically position themselves along the political spectrum to maximize voter support. This concept is often compared to marketing strategies, where brands differentiate themselves to capture consumer markets [7]. However, unlike commercial positioning, which centers on shaping consumer perceptions, political parties must navigate complex, multidimensional ideological spaces to appeal to diverse voter groups by developing distinct policy positions and value propositions.

Despite the utility of spatial models, their limitations in conceptualizing party positioning have been widely acknowledged. First, political competition involves more than just positional issues. It also includes valence dimensions—such as perceived competence and broader goals like economic prosperity—that complicate voter evaluations [2]. Second, the single left-right spectrum obscures key distinctions between economic and social policies, as cross-national studies show parties adopting hybrid positions [3]. Third, non-positional factors like party image and leadership credibility often weigh more heavily on voter preferences than specific policies, and voters may not perceive strategic platform shifts [4]. Finally, cognitive biases and media framing further shape—and sometimes distort—voter perceptions, challenging the idea that behavior aligns solely along spatial dimensions. To address these complexities, scholars stress incorporating contextual factors such as issue salience and framing effects when analyzing party positions, as underscored by Jacoby’s foundational research [8–9].

The formation of voter perceptions regarding party positions is influenced by diverse information sources, including political discourse [10], policy realignments [11–12], coalition formations [13–14], leadership changes [15], and issue prominence [16]. These varied influences help voters build mental frameworks to interpret party positions, often relying on party documents such as manifestos or legislative speeches [17]. However, textual analyses of these documents frequently yield inconsistent results. This is due to irrelevant dimensions and the difficulty of separating rhetorical from substantive content, which results in a lack of standardized measures for assessing party similarity.

This study adopts a mixed-methods analytical framework centered on positioning maps derived from spatial models, which geometrically—often using Euclidean metrics—represent similarity within a policy space [18–20]. However, spatial models usually assume symmetric similarity and often neglect contextual or feature-specific dynamics. To address this limitation, the study integrates Tversky’s feature matching model [21], which compares shared and unique attributes with asymmetric weights, capturing the varying importance of features in voter perceptions. By combining these complementary approaches, this research advances theoretical understanding of party similarity and provides a robust methodological foundation for analyzing complex voter perceptions.

### Political cleavages, party competition, and voter alignment: The case of Taiwan

In recent years, escalating U.S.-China tensions have emerged as a critical challenge to the international order, with much of the focus concentrated on the contentious China-Taiwan relationship. As a key factor in cross-strait relations, Taiwanese perceptions of China remain central in shaping discourse on the island’s future, particularly as Chinese leadership continues to emphasize Taiwan’s “recovery” as a vital element of national rejuvenation [22]. Notably, despite Taiwan’s growing economic dependence on mainland China over recent decades, this economic interdependence has not translated into close political alignment. This phenomenon is difficult to explain using the commonly used political theoretical frameworks of realism and liberalism [23], which suggests that Taiwanese political attitudes are likely more nuanced and multifaceted. Moreover, mounting external pressures from China—including diplomatic isolation efforts, military posturing, and economic coercion—have further intensified internal political divisions. Clark and Tan [24] highlight a paradox within Taiwan’s political landscape, where internal polarization fails to fully capture the complexity of public opinion: while elite-level politics have become increasingly polarized—especially on national identity and cross-strait relations—voters tend to hold more moderate views. They attribute this divergence to the rise of ideological activism, salient cultural and identity issues, and the decline of catch-all parties. This dynamic complicates Taiwan’s political environment and underscores the importance of examining voter alignment.

The gap between elite polarization and voter moderation has created opportunities for new parties to emerge. Within Taiwan’s evolving political landscape, the rise of “small parties” is reshaping the political scene [25]. These parties expand political discourse by addressing issues beyond traditional national or ethnic identity concerns. Zons [26] categorizes these emerging parties into “truly newly established” parties and those formed through splits within existing parties, reflecting a shift in voter priorities toward broader societal concerns. Taiwan’s complex external geopolitical environment, combined with its multi-party system—including some opposition parties that advocate relatively conciliatory policies toward China—makes it a critical case for political analysis. Its competitive and fluid party landscape, characterized by frequent realignments and the presence of multiple parties, offers an ideal setting to explore how voters perceive party similarity. This includes investigating whether these perceptions are limited to a single ideological dimension, as well as testing multidimensional models of party positioning.

Building on the evolving party landscape and voter dynamics, it is important to recognize that Taiwan’s predominant political cleavage centers on national identity and the issue of independence versus unification with China [27]. Historically, the independence-unification issue has overshadowed other political topics such as social justice and economic development [28]. In recent years, public opinion on this matter has fluctuated significantly, influenced by both external and domestic factors [29]. Within this context, Lin, Wu, and Charm [30] categorize Taiwanese voters’ cross-strait positions into symbolic and pragmatic types. The symbolic type involves firm, unconditional support or opposition to Taiwan’s independence regardless of potential Chinese aggression, driven by identity, cost-benefit considerations, or both. The pragmatic type, in contrast, reflects conditional support depending on favorable external circumstances, such as the absence of Chinese military threats. They observed a shift toward more symbolic and uncompromising positions, suggesting that Beijing’s actions—whether friendly or hostile—have diminished influence on Taiwanese voter attitudes. Complementing this perspective, Liu and Li [23] argue that cross-strait relations are socially constructed through interactions between both sides. From a constructivist viewpoint, Beijing and Taipei are not necessarily irreconcilable adversaries but need to shift from a dialogue of confrontation or conflict to a dialog of peace. Their study shows that, although the younger generation in Taiwan holds politically hostile attitudes toward China, they nonetheless support trade negotiations and peaceful dialogue.

In other words, the rise in symbolic political stances simultaneously reflects voters’ strong expression of identity and heightened emotional vigilance in response to China’s military threats. Nevertheless, it also coexists with a certain degree of desensitization or emotional numbness among some voters, resulting from prolonged military pressure. In this context, frequent military provocations have led to psychological habituation, causing some voters to become desensitized and lose interest in highly polarized political issues. Additionally, fluctuating international attention and Taiwan’s increasingly diverse society have contributed to voter fatigue and skepticism toward the independence-unification debate, with some perceiving it as overly politicized, a pseudo-issue, or a debate disconnected from realities. As a result, some have begun to regard the independence-unification debate as less salient, potentially shifting their focus toward more immediate concerns related to personal well-being and socioeconomic livelihood. This observation prompts the present study’s discussion of the possibility that a second dimension exists in the literature and among Taiwanese voters’ political perceptions beyond the unification–independence issue.

Since democratization in the 1990s, Taiwan has experienced significant political and social transformations that have deeply shaped individual and collective identities. This process not only initiated political reforms but also accelerated Taiwanization (or indigenization), fostering a distinct Taiwanese identity and nationalism [31]. After more than half a century of cross-strait separation, Taiwanese nativism has also emerged, emphasizing local culture, values, and lived experiences [32]. As Liu and Li [23] point out, different generations in Taiwan have undergone distinct socialization processes. Since the separation, Taiwan has developed into a multi-party democracy, while mainland China remains an authoritarian single-party state. This evolving Taiwanese identity has contributed to growing public resentment toward Beijing, driven not only by opposition to unification but also by the younger generation’s weakened cultural affinity and institutional identification with China. Conversely, advocates of unification often depict supporters of Taiwanese identity as neglecting their historical roots or as economically dependent on the mainland China, using emotional appeals to challenge Taiwan’s distinct identity.

Democratization in Taiwan has also expanded civic space, enabling diverse social movements to flourish. A notable example is the 2019 legalization of same-sex marriage—the first in Asia—which signifies progressive internal social transformation emphasizing individual freedoms and rights alongside continuing debates over national identity. At the same time, the island’s two major parties reflect their identity divisions: the Kuomintang (KMT) generally favors unification, the Democratic Progressive Party (DPP) supports independence, and non-partisan or centrist voters typically prefer maintaining the status quo [33–34], in which blue is the representative color of the KMT, while green represents the DPP. Wang [35] observed no clear overall polarization trend among Taiwanese voters regarding cross-strait relations. However, when partisans become polarized, moderate supporters may leave one or both parties, exacerbating partisan divides, whereas if non-partisans are attracted to one of the major camps, the partisan distribution shifts toward the center. His empirical evidence confirms that these dynamics have materialized in Taiwan, shaping its current political landscape.

Liu and Tsai [36] provide another empirical evidence supporting the development of partisan-motivated reasoning in Taiwan. Their study challenges the conventional view that “independent voters” are genuinely neutral or unaffiliated with any political party. Instead, many of these self-identified independents are actually “closet partisans” who conceal their true party affiliations during surveys. By 2017, over half of Taiwanese voters identified as non-partisan or independent voters according to the Taiwan National Security Survey [35,37], a record high attributed to public dissatisfaction with major parties, electoral reforms favoring a two-party system, and generational shifts as younger voters increasingly reject traditional party affiliations [37]. This trend prompts critical investigation into how independent voters perceive political party images and evaluate party similarities, with particular attention to different voter groups in this study.

The above literature review reveals that Taiwan currently has a significant number of voters who are discontented with the major parties but continue to value and uphold democratic ideals, creating opportunities for new political parties to emerge and gain support. In other words, the intricate interplay of Taiwanese identity, economic development and livelihood concerns, as well as democratization, coupled with the notable presence of closet partisans and non-partisan voters, has introduced new options in Taiwan’s party politics. While engagement across the Taiwan Strait encourages dialogue, it also exposes a deep divide between Taiwan and China, intensifying anxieties over identity and fostering exclusionary sentiments. This heightens social polarization as differing opinions and interests become more pronounced across society, contributing to elite resentment—conditions often associated with the rise of populist movements [38]. Indeed, populism is frequently characterized by negative emotions directed toward elites and out-groups [39]; yet it remains an inherent feature of democratic politics, reflecting the tensions and contradictions embedded within democratic systems.

This study provides new empirical evidence by quantitatively analyzing voter self-reports to reveal how political parties are perceived and differentiated in Taiwan. It adopts a mixed-methods, voter-centric approach specifically tailored to Taiwan’s distinctive political context. The research design enables a more nuanced and comprehensive analysis of voter perceptions and party competition, offering a replicable framework that advances current studies on electoral dynamics in emerging democracies. Such insights are crucial for deepening our understanding of political polarization and informing strategies to mitigate its effects, ultimately contributing to democratic consolidation.

### Research design and method

This study conducted an online survey from November 1, 2021, to December 10, 2021, and entrusted Shanshui Public Opinion Research Company, a professional polling firm in Taiwan, to execute the survey. During this period, Taiwan was engaged in discussions about a series of national public referendums, which included four cases covering diverse policy issues such as environmental protection, energy, food safety, international trade, and the referendum mechanism itself. The following year, national local elections were scheduled, providing a crucial opportunity for smaller parties and emerging political forces to gain traction and potentially reshape the political landscape.

The study protocol and methodology were reviewed and approved by the MacKay Memorial Hospital Institutional Review Board (Approval number: 20MMHIS171e). Informed consent was waived by the IRB due to the anonymous nature of the internet-based survey, which collected data from Taiwanese voters without personally identifiable information. Participants were randomly sent Short Message Service (SMS) messages with unique questionnaire IDs, which they used to access the online survey platform. To ensure that each participant could only submit one response, the backend system verified consistency between the mobile number submitted for the public lottery after completing the survey and the number used for SMS transmission.

Given the survey’s 20-minute duration, interviewers were not feasible. Instead, attention-check items were included to filter out illogical responses. For instance, these items checked for inconsistencies such as providing illogical responses to reverse-coded questions, or rating party support outside the range between their “most supported” and “least supported” parties. These checks allowed me to identify and exclude participants who appeared to be inattentive.

The survey yielded a total of 4,012 responses, including some that were incomplete or failed attention checks. Among these, 1,340 questionnaires were fully completed and qualified, resulting in a qualification rate of approximately 33.4%. Although this qualification rate may seem relatively low, it is essential to note that this research focus is on obtaining high-quality, complete samples rather than solely on response rates. The 1,340 fully completed and qualified questionnaires provide sufficient data support for meaningful analysis. Furthermore, these samples can be weighted to represent the broader population, thereby enhancing the generalizability of the findings.

Notably, this sampling design is identical to that of another study, which also utilized the same 1,340 samples for a different topic and was published in a Taiwanese TSSCI journal [40]. To ensure the sample’s representativeness, I applied the multivariate raking method for post-stratification weighting, using the latest demographic statistics from the Ministry of the Interior as the population parameters, as detailed in Appendix 2 [40]. Following this nationally representative adjustment, the weighted sample of 1,340 responses was confirmed to be consistent with the population structure in terms of gender, age, educational level, and place of residence, as verified by chi-square tests (*p* > 0.05). By comparing the weighted sample calculations to the party-list ballot shares of the three major parties in the 2020 legislative elections, I found that the estimates closely approximated actual results [40]. This indicates that the weighted samples possess a certain level of reference value and provide a credible foundation for making overall estimates of Taiwanese voter behavior. The data underlying the findings described in this manuscript are available as supplementary information. These include the questionnaires (S1 File) and original survey results (S1 Table) in Chinese, which can be accessed directly from the supplementary materials.

This study adopts an open-ended questionnaire that allows respondents to freely identify and describe clusters of similar parties in Taiwan, capturing alternative dimensions voters use to express their overall impressions. It also emphasizes the importance of understanding how voters’ perceptions of smaller parties are shaped in relation to mainstream parties. Specifically, it investigates whether small parties possess core characteristics that are consistently recognized across voter groups, or whether these perceptions are conditional, varying depending on specific voter segments.

The data analysis starts with an attempt at Multiple Correspondence Analysis (MCA), a multivariate statistical technique [41–42], to analyze categorical data from open-ended survey questions. MCA transforms datasets into a multidimensional Euclidean space, enabling the examination of relationships between multiple categorical variables and subjects such as candidates or political parties, thereby effectively constructing positioning maps.

Inspired by Nishisato’s example involving the classification of 35 animal names by 15 university students [42] (p. 147), this study adopts a similar approach by asking respondents to categorize Taiwanese political parties based on their perceived similarities. Nishisato’s example illustrates how MCA can provide valuable insights into classification processes. Firstly, it reveals relational groupings among animals, such as categorizations based on habitats (terrestrial, marine, aerial, etc.), which yield relevant classification clusters. Secondly, it examines the classification methods used by students and their interrelations, showing how different students may employ distinct approaches to categorize animals, yet their results may exhibit correlations (e.g., Student A’s second group might correspond to Student B’s third group). Lastly, it investigates the relationship between the students’ diverse classification methods and the interrelations among the animals themselves.

In the initial step of this study, open-ended questions were employed to capture the images of party groups and the criteria used for grouping as follows:

1 **Grouping Similar Parties:** Please group the political parties in Taiwan that you perceive as similar in their stances on various issues. If you believe a party is distinct from others, you can place it in a separate group. How many groups do you think these parties can be categorized into? (Please fill in a number greater than 1) The parties listed include: *Kuomintang (KMT), Democratic Progressive Party (DPP), New Party (NP), People First Party (PFP), Taiwan Solidarity Union (TSU), Green Party Taiwan (GPT), New Power Party (NPP), Taiwan Statebuilding Party (TSP), Taiwan People’s Party (TPP).*
2 **Basis for Grouping:** Please briefly describe the criteria you used for grouping these parties. (*Open-ended question, mandatory response.*)
3 **Parties in Each Group**: Which parties do you include in each of the groups you identified? (*Select party names.*)
4 **Group Description (One by One)**: For each group, please describe the characteristics or common traits that led you to group these parties together. *(Open-ended question.)*

The survey includes nine political parties that were well-known to the public and appeared on the 2020 Taiwanese legislative party-list ballot. The smaller parties are generally associated with the broader pan-blue or pan-green camps. Additionally, the TPP is commonly referred to as the third force, or the “white camp.” The prompt, “Many political parties have similar stances on various issues...,” encourages respondents to consider the similarities and differences among parties. After collecting the survey data via online platforms (e.g., smartphones, tablets), the open-ended responses can be analyzed using text mining techniques [43]. This involves categorizing the criteria for party grouping and integrating the descriptions of each party cluster.

In the second step, I use MCA to create a positioning map that identifies similarities among political parties. MCA places each party as a point in a multidimensional space, where distances reflect their similarity. This method uncovers the underlying structure of respondents’ classification data, allowing a nuanced understanding of party relationships and the dimensions shaping their positions. This phase of the research produces three main outcomes: a party positioning map, party clusters with descriptions, and insights into party relations and competitive dimensions.

This study utilized R programming language packages, including FactoMineR and ExPosition, to create positioning maps. Initially, I employed the MCA function from the FactoMineR package to perform MCA. Subsequently, I used the factoextra package to generate visualizations of the results.

Regarding keyword extraction from responses to open-ended questions, I briefly describe the criteria used for grouping parties as follows. The text preprocessing involved several steps: removal of symbols and stop words—common function words in Chinese phrases that carry little substantive meaning or contextual importance (e.g., 的, 了, 是)—is a necessary step to reduce noise and enhance the efficiency of the analysis, and customization of dictionaries to include political terms specific to Taiwan. I utilized the JiebaR package for text segmentation and iteratively refined the dictionary based on word frequency statistics, ensuring consistency across three rounds of processing. The top 20 most frequent terms stabilized after these iterations (S2 Table). To ensure the clarity of segmentation, I implemented the following measures: 1. Customized dictionary: Included Taiwan-specific political terminologies, which were refined based on word frequency statistics from each round of processing. 2. Standardization: I unified similar concepts by converting terms like “泛藍 (pan-blue)” and “泛綠 (pan-green)” to “藍綠 (blue-green)”, and standardizing “台獨 (Taiwan independence/Taiwanese independence)” as “獨派 (pro-independence camp)”. 3. Filtering: Retained only words with a length greater than one character. I extracted the top 20 most frequent terms for descriptive analysis. However, some keywords in the raw data, such as “舔不舔 (tian bu tian)”, may have been overlooked and fragmented into disjointed words due to their colloquial nature or dialectical variations, potentially reflecting limitations of the JiebaR segmentation tool. In Taiwan’s political discourse, *tian bu tian* (舔不舔, literally “lick or not lick”) often refers to whether parties excessively ingratiate themselves with the Chinese Communist Party or pro-China forces, reflecting a politically charged expression of sycophancy or flattery. Nevertheless, by focusing on the top 20 most frequent terms, I ensure that the analysis emphasizes high-frequency terms, which helps maintain the validity of my research inferences.

In the third step, I identify which political parties are distinctly different. While spatial models based on Euclidean distance assume symmetrical relationships, cognitive psychology—particularly Tversky’s feature matching model [21]—shows that similarity can be directional. For example, “a resembles b” does not imply “b resembles a” equally. Nebus and Celo [44] experimentally confirmed this asymmetry, demonstrating that perceived distances depend on both entity characteristics and observer judgments.

Tversky [21] also explored dissimilarity judgments using two groups of 23 participants each. One group was asked to judge the difference between countries p and q, denoted as d(p, q), while the other group judged the difference between q and p, denoted as d(q, p), with q being a more prominent country. The results showed that the average of d(p, q) was significantly higher than the average of d(q, p) across all pairs, further highlighting the asymmetry in comparisons involving prominent and less prominent entities. Applying this concept to political parties, the study utilizes similar questioning techniques to assess respondents’ perceptions of party differences. For instance, I consistently place the major Taiwanese political parties at the end of the questions, and ask respondents to rate their agreement with statements such as:

1 *How much do you agree that the Taiwan Statebuilding Party differs from the Democratic Progressive Party on many political stances? (Scale: 0–10)*
2 *How much do you agree that the Taiwan People’s Party differs from the Democratic Progressive Party on many political stances? (Scale: 0–10)*
3 *How much do you agree that the New Power Party differs from the Democratic Progressive Party on many political stances? (Scale: 0–10)*
4 *How much do you agree that the New Power Party differs from the Kuomintang on many political stances? (Scale: 0–10)*
5 *How much do you agree that the Taiwan People’s Party differs from the Kuomintang on many political stances? (Scale: 0–10)*
6 *How much do you agree that the New Party differs from the Kuomintang on many political stances? (Scale: 0–10)*

After integrating all respondents’ answers, I can compare the overall impressions of Taiwanese voters with different party inclinations, providing insights into how various conditions influence perceptions of party differences. To assess voting party inclinations, respondents were asked: “Which of the following best describes your voting party inclination? (01) Strongly pan-blue, (02) Pan-blue (泛藍), (03) Third force, (04) Pan-green (泛綠), (05) Strongly pan-green, (06) No specific party inclination.” This approach allows me to explore how different political orientations influence voter perceptions.

In the final step, I applied Tversky’s feature matching model [21] to obtain detailed descriptions of specific party images. Tversky illustrated that while Jamaica and Cuba are similar due to geographical proximity, and Cuba shares similarities with the Soviet Union because of comparable political systems, Jamaica and the Soviet Union show no similarity. Tversky proposed a matching function s(a,b)=F (A∩B, A − B, B − A) to calculate similarity between a and b, where F depends on three components: A∩B, features common to both; A − B, features unique to a; and B − A, features unique to b. Given the rise of third-force parties in Taiwan, understanding how voters perceive these parties is crucial. I focus on comparing them with the two major parties by identifying and analyzing shared features through text analysis to uncover key party images that shape voter perceptions.

This step employed the widely used Latent Dirichlet Allocation (LDA) method, a generative probabilistic model that is commonly utilized in natural language processing and text mining [43,45]. LDA has been widely applied in various fields, such as identifying themes in large-scale textual datasets, extracting key topics from social media posts, and analyzing survey responses. In this study, LDA was employed to analyze the open-ended responses from specific voter groups, following segmentation using an R-based Jieba program for Chinese text preprocessing, as described previously. LDA explores co-occurrence relationships between words, thereby revealing deeper semantic structures compared to simple word frequency statistics. Applying this approach through topic modeling, the study aimed to uncover the most salient latent topics and extract important keywords that encapsulate the perspectives and concerns of Taiwanese voters.

## Results

**Fig 1:**
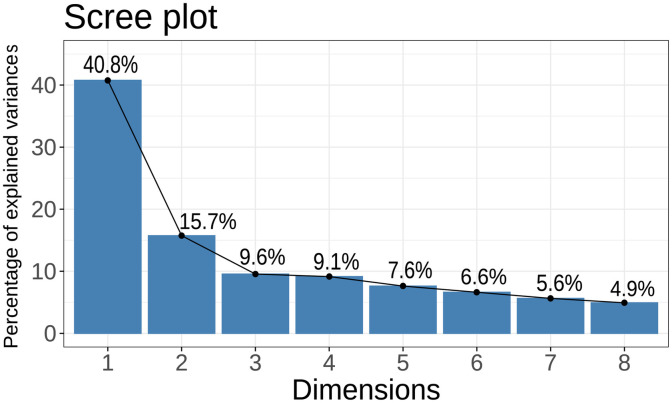
Scree Diagram for MCA.

As shown in Fig 1, this study utilized MCA to explore the perceptions of political parties among Taiwanese voters. A scree plot was generated to visualize the percentage of variance explained by each dimension, guiding the selection of dimensions. In MCA, the scree plot is used to display the total inertia explained by each dimension [40–41], which helps researchers determine whether the party images are confined to a single dimension. To determine the optimal number of dimensions, I employed a combination of the elbow method and visual inspection of the scree plot. The elbow method involves identifying the point at which the rate of increase in explained variance begins to slow down, indicating the optimal number of dimensions to retain [41], Specifically, I considered dimensions that collectively explain more than 50% of the variance as sufficient for capturing the underlying structure of party perceptions. Notably, the first dimension accounts for 40.8% of the variance, suggesting that it plays a significant role in explaining how Taiwanese voters perceive political parties. The second dimension also explains a partial portion of the variance, at 15.7%. Furthermore, Fig 1 indicates that dimensions beyond the second contribute to the explanation, albeit to a lesser extent. This suggests that multiple dimensions are involved in shaping the voter perception of political parties in Taiwan.

Upon analyzing the top 20 keywords used to briefly describe the criteria for grouping parties, I observed that the term “blue-green” was the most frequently mentioned, accounting for 11.03% of all references among the top 100 keywords. This prominence reflects a general trend, as the percentage of references for keywords beyond the top 100 is negligible and thus can be disregarded. Following “blue-green, ” other notable terms included “ideology” (6.25%), “pro-China” (5.38%), “Taiwan” (2.90%), “unification-independence” (2.07%), and “cross-strait” (1.22%). These findings suggest that the blue-green, unification-independence issue, or cross-strait relations, remains a dominant factor in describing the criteria used for grouping parties. The appearance of keywords such as “color” (3.49%) and “blue-green-white” (2.70%) further underscores the salience of party positioning in Taiwanese voters’ perceptions, particularly regarding the TPP. Founded by former Taipei Mayor Ko Wen-je and symbolically linked to his informal“White Force” group, which emphasizes open governance, the TPP had established a notable presence in Taiwanese politics by late 2021, during the period of the survey.

Interestingly, traditionally emphasized aspects such as economic policy, welfare, environmental issues, and corruption did not feature prominently among the top 20 keywords. Instead, phrases like “whether a party gets things done” (1.36%) and “policies and issues” (3.33%) were highlighted, reflecting voters’ increasing prioritization of administrative performance and effective governance over traditional ideological divisions. Additionally, keywords like “media or impression” (2.78%) and “feeling” (1.65%) indicate that media framing effects and subjective impressions also play a partial role in shaping voter perceptions of political parties.

These findings are consistent with prior research and indicate that traditional ideological cleavages continue to play a pivotal explanatory role in structuring voter perceptions. This divide is best captured by the first dimension in Fig 1. At the same time, the growing importance of factors such as administrative performance, media framing, and other contextual influences is becoming increasingly evident in how voters perceive party groupings in Taiwan. This emerging pattern suggests a broader trend in which voters are placing greater emphasis on effective governance and responsive leadership when evaluating solutions to pressing domestic challenges.

**Fig 2:**
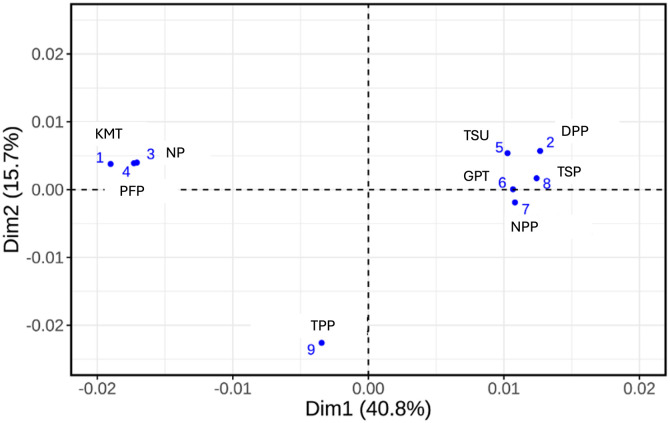
Voter Perception of the Positioning of 9 Political Parties in Taiwan.

Furthermore, the results of MCA can be visualized using a scatter plot to illustrate the positioning of political parties. By analyzing the distribution of points in the graph, I can understand the distance relationships and similarity levels between different parties. According to Fig 2, when respondents were asked to group similar parties together, several important clustering phenomena emerged. Fig 2 provides a basis for understanding the possible meanings of these dimensions.

Upon initial observation of Fig 2, the first dimension appears to divide the parties into two major groups: Group 1, the pan-blue camp, consisting of the KMT, NP, and PFP; and Group 2, comprising the TSU, GPT, NPP, TSP, and DPP. The TPP, with a negative value on the first dimension, seems to lean towards Group 1. Notably, despite the GPT’s unique ideological characteristics, it is generally categorized as part of the pan-green coalition, Group 2, by Taiwanese voters, primarily due to its name.

Upon closer inspection of Fig 2, the first dimension appears to represent the traditional blue-green and unification-independence divide, reflecting the distinct stances of major parties on cross-strait relations. The second dimension, however, captures aspects that diverge from these traditional frameworks. Notably, only the NPP and TPP exhibit negative values on this dimension, whereas most traditional blue-green parties display positive values. This dichotomy in values highlights the divergent positions not only between the blue-green camps but also underscores the distinctiveness of third-force parties like the NPP and TPP, which differentiate them from the traditional political landscape. The presence of the second dimension suggests that parties located in the third and fourth quadrants may transcend the traditional blue-green and unification-independence frameworks, presenting new party images. The GPT, with a value close to zero in the second dimension, does not align with this trend. If only the first dimension is considered, the NPP would necessarily be categorized within the pan-green camp.

### Are new competitive political dimensions emerging?

In this study, I employed randomization in distributing questionnaires (S1 File). Approximately half of the respondents (727) received a list of nine major political parties in Taiwan, while the other half (613) were presented with an expanded list that included three additional smaller parties: the Taiwan Penghu Party, the Joyful Island Alliance, and the Chinese Unification Promotion Party. These smaller parties were selected from the 2020 legislative party-list ballot as well. By using this list experiment design, I aimed to assess the absence of design effects [22]—specifically, whether the presentation of additional parties in the list influences respondents’ perceptions and categorizations of the original nine prominent parties beyond the intended effects of the experiment. This allowed us to explore how exposure to a diverse range of parties might shape voters’ perceptions and categorization of these parties. The findings indicate that regardless of whether respondents were shown by nine or twelve parties, their perceptions of the nine prominent parties remained consistent as shown in Fig 2. This consistency between the half-subsample and the full sample serves as robust evidence for the reliability of the MCA method, demonstrating stable party positions in the mindsets of Taiwanese voters.

As mentioned, the second step of the data analysis involved examining respondents’ self-reported descriptions of the criteria used for grouping parties, which highlighted the enduring significance of traditional blue-green and unification-independence divides in Taiwanese voters. Additionally, I complemented this approach with another method to further understand party groupings. In the context of mapping party positions, researchers often seek to understand the underlying logic of different dimensions. To achieve this understanding, I can infer the most important variable categories contributing to the first and second dimensions by analyzing respondents’ descriptions of party groups. I utilize the squared correlations between variable categories and dimensions (cos^2^) as a criterion to determine the contribution of each variable category to these dimensions [41].

Table 1 represents the principal elements associated with each dimension in Fig 2. Since each respondent can be considered a unique classification variable, I can deduce which variable categories correspond to important party group characteristics as provided by respondents. As each respondent categorizes political parties into at least two distinct groups, Table 1 employs the notation ‘s945.1’ to denote the first group description provided by respondent #945, and ‘s406.4’ to indicate the fourth group description from respondent #406, with analogous coding applied to all entries.

**Table 1:** Top 20 Ranked by Contribution.

Dim 1	Group Description	Dim 2	Group Description
s945.1	Not pro-China	s406.4	Political newcomer
s1298.1	Supporting high-ranking CCP officials doing business on the mainland	s945.3	Unprincipled and opportunistic
s1298.2	Drawing a clear line with the mainland	s863.3	Centrist force
s406.2	Split between brothers	s951.3	Having ideals
s945.2	Too pro-China	s852.2	Caring about hard-working laborers
s642.1	More peaceful	s1199.2	Unconventional
s863.1	Pan-blue	s1270.2	Both pro-China and anti-China factions
s1270.3	Anti-China	s226.2	Acceptable
s951.1	Same faction	s642.2	Desire to change
s1031.1	Blue	s1031.3	Others
s1270.1	Somewhat pro-China	s874.3	Disorganized
s874.1	Each doing their own thing	s498.3	Need to work harder
s406.1	Circle of close friends	s295.3	Poor leadership
s1326.1	Blood is thicker than water	s1112.3	Will exposure without a central idea
s1326.2	The same political color	s173.3	Centrist with a leaning towards local characteristics
s863.2	Pan-green hypocritical party	s1205.3	Light blue and light green
s315.1	Uninspired	s497.2	Unable to express the meaning of representation
s296.2	Only caring about the Communist Party and neglecting Taiwan	s795.3	Relatively casual
s1232.3	Taiwan independence and nation-building	s486.1	The party is relatively larger
s95.1	Same ideology	s486.2	More able to think for people

Table 1 demonstrates that the first dimension of party positioning is characterized by stances on cross-strait relations and blue-green party identification. This dimension is represented by the top 20 contributing variable categories, which include descriptors such as “not pro-China,” “somewhat pro-China,” “too pro-China,” “pan-green,” and “pan-blue.” These terms reflect the relatively prevalent party group characteristics associated with the first dimension depicted in Fig 2. The second dimension, however, does not clearly align with conventional issues like social distribution, fairness, wealth disparity, or generational differences. Instead, it serves to distinguish parties that transcend the conventional blue-green divide, particularly highlighting the emergence of new third forces, NPP and TPP.

In Dimension 2 of Table 1, the emerging political parties exhibit diverse characteristics, signaling a departure from traditional political alignments. The rise of new parties in Taiwan has introduced a fresh dimension of political diversity, offering voters a wider range of choices and voices. Terms such as “political newcomers,” “desire to change,” “unconventional,” “opportunistic,” “without a central idea,” “concerned about hard-working laborers,” and “more able to think for people” capture the complexity and diversity of these parties. From a populist perspective, these descriptions reflect “people-centered” and “anti-elitist” tendencies, core elements of populism. Populists often perceive existing political systems as dominated by elites who fail to represent the people’s will genuinely, thus advocating resistance against the established order.

Additionally, phrases such as “light blue and light green,” along with references to “both pro-China and anti-China factions,” highlight the nuanced and complex nature of these emerging parties. The rise of“centrist forces emphasizing local characteristics” further diversifies Taiwan’s political spectrum, especially its connections to Taiwanese nationalism and nativism, which underscores local values while also accommodating moderate supporters from both the blue and green camps.

Through these research explorations, it becomes evident that the phrases used to describe emerging parties reveal at least a second dimension of political diversity and social value transformation in Taiwan that cannot be overlooked. This evolving political dimension can be conceptualized as “Diverse Political Engagement” or “Diverse Political Ecology.” These terms encapsulate the multifaceted roles that new and unconventional parties play in broadening electoral choices and reflecting the heterogeneous concerns of voters, including democratic ideals, economic development, livelihood issues, and localized identity, while fostering a more inclusive and pluralistic political environment. Unlike traditional political frameworks structured by binary divides such as blue-green or unification-independence, this emerging political ecology introduces alternative pathways of representation that better reflect growing political diversity. This shift signals a profound transformation in Taiwan’s political landscape, meriting further in-depth investigation and longitudinal study.

### Party similarity through feature matching

Fig 2 illustrates that the TPP is positioned on the left side of the party positioning map; however, when assessed using the Euclidean distance approach, the TPP is nearly equidistant from both the KMT and DPP, suggesting an ambivalent role regarding independence-unification issue. To further understand the differing perceptions of party positioning among voters based on their voting preferences, I surveyed respondents about their party inclinations, as mentioned previously. The overall statistical distribution revealed that 28.2% identified with the pan-blue camp (including strongly pan-blue), 28.8% with the pan-green camp (including strongly pan-green), 8.9% identified as third force voters, and 34.2% indicated no specific party inclination. Since third force voters are relatively few, this study combines them with individuals who have no specific party inclination to create a single group labeled “Voters Outside the Traditional Blue-Green Spectrum.” Non-parametric statistics are then employed to avoid assumptions of equal variance among the groups.

This study employed the Kruskal-Wallis test to examine differences in median values among three groups. As shown in Table 2, there was a consensus regarding statements such as “The TSU differs from the DPP on many political stances” and “The NPP differs from the DPP on many political stances.” However, other comparisons exhibited statistically significant differences (*p* < 0.001). Table 2 suggests that perceptions of similarity among voters can vary based on different party inclinations. For instance, while both the TSU and NPP are generally perceived as leaning towards the pan-green camp in Fig 2, pan-green voters distinctly view these parties as different, with the TSU being seen as more similar to the DPP. Furthermore, the distinctions between pan-blue and pan-green supporters regarding their views on the TPP in relation to the KMT and DPP warrant deeper discussion. Table 2 indicates that pan-green supporters perceive the TPP as slightly leaning towards blue but distinct from green, whereas pan-blue supporters view it as situated in between.

**Table 2:** Pairwise Comparison of Political Parties by Voter Party Inclination.

	Pan-blue		Pan-green		Voters outside the traditional blue-green spectrum		KW test
	(n = 378)		(n = 385)		(n = 577)		
Pairwise Comparison	Median	IQR	Median	IQR	Median	IQR	*P*-value*s*
Is TSP different from DPP?	3.62	4.00	3.00	3.00	4.00	3.00	0.140
Is TPP different from DPP?	5.00	3.00	7.00	4.00	5.00	3.00	<0.001
Is NPP different from DPP?	3.00	5.00	4.00	2.13	4.00	3.00	0.028
Is NPP different from KMT?	8.00	4.00	7.00	3.00	7.00	3.00	<0.001
Is TPP different from KMT?	5.00	2.00	4.00	4.00	5.00	3.00	<0.001
Is NP different from KMT?	5.00	3.00	2.00	5.00	4.00	4.00	<0.001

Notes: IQR = interquartile range, which stands for the difference between the third quartile (Q3) and the first quartile (Q1) of data distribution.

The observed deviations in voter perception may stem from a tendency among some voters to instinctively create “in-group” and “out-group” classifications. Voters aligned with either the pan-blue or pan-green camps are more likely to hold favorable views of similar parties within their in-group while expressing skepticism towards out-group parties. In contrast, independent voters tend to exhibit less pronounced distinctions along these lines. Reflecting on past trends and current developments, the rise of third-force movements in Taiwan has gained significant international attention, especially given recent events such as the substantial defense budget cuts proposed by the legislative opposition in 2025 [46]. This study from 2021 has already shown that the TPP is perceived by pan-green supporters as distinct from the pan-green camp, which complicates potential cooperation between the TPP and DPP. This differentiation may provide an opportunity for the TPP and KMT, both major opposition parties, to reinforce their cooperation.

Table 3 employs the feature matching model to summarize the characteristics of major small parties in Taiwan. Notably, the TPP stands out, with 22.53% of respondents classifying it as a distinct group—the only party to be independently categorized by voters. This unique classification underscores the TPP’s establishment of a clear and separate identity within Taiwan’s party system. Such a distinction aligns with Zons’ categorization of political parties [26], which differentiates between “truly newly established” parties and those formed through splits from existing organizations. The TPP exemplifies the former, demonstrating its emergence as a genuine new political force in Taiwan’s evolving multi-party landscape.

**Table 3:** Voter Perceptions of Major Small Parties in Taiwan.

Party	Clustering content	Main features	Intersection features with DPP	Intersection features with KMT
**TPP**			Native ethnic groups, Large online presence, Action-oriented, Use of online campaigns, Localism	Friendly with the CCP, Conservative, Overlapping voters, Pro-China faction
1	TPP	1.Neutral stance, Lack of clear core values, Ko Wen-je’s party		
2	KMT + NP + PFP + TPP	2.Pro-China, Pan-blue, Pro-unification		
3	NPP + TPP	3.Third force, No clear blue-green stance, Focused on people’s livelihood		
**TSP**			Pan-green camp, Democratic Taiwan, Taiwan first, Anti-China and defend Taiwan	Dislike them all politicians
1	DPP + TSU + GPT + NPP + TSP + TPP	1.Localism, Taiwan independence, Pan-green		
2	DPP + TSU + TSP + TPP	2.Taiwan first values, Anti-China, Pro-U.S.		
3	TSP + TPP	3.Preserve Taiwan’s culture, Taiwan sovereignty awareness, Concern for democracy and freedom		
**NPP**			Human rights awareness, Emphasizing individual Freedom and democracy, Social reform, Respect for public opinion	Opposition party, Balancing various interests and needs, Focused on public opinion
1	DPP + TSU + GPT + NPP + TSP + TPP	1.Local, Pro-independence, Pan-green		
2	NPP + TPP	2.Third force, No clear blue-green stance, Focused on people’s livelihood		
3	DPP + TSU + NPP + TSP + TPP	3.Democratic values, Opposed to KMT, Pan-green		
**PFP**			Focus on people’s needs, Incorporates traditional elements, Local	Pro-China stance, Support for cross-strait unification, Pan-blue, Close to CCP, Lack of resistance to CCP
1	KMT + NP + PFP + TPP	1.Pan-blue, Conservative, Pro-unification		
2	PFP + TPP	2.Party leader with charismatic leadership, Courageous in speaking for the people, Politically neutral ideology		
3	KMT + PFP + TPP	3.Pro-China, Pan-blue camp, Policy views similar to KMT		
**NP**			Focus on social issues, Strong ideological stance	Pro-China, Pro-unification stance, Conservative traditional values, Skilled in political maneuvering
1	KMT + NP + PFP + TPP	1.Pan-blue, Conservative, Pro-unification		
2	NP + TPP	2.Pro-unification, Red, Authoritarian		
3	KMT + NP + TPP	3.Pro-China, Pan-blue, Conservative party		

This distinct group is characterized by its neutral stance, lack of clear core values, and association with Ko Wen-je. Additionally, Table 3 highlights that the TPP shares some overlapping voters with the KMT while its intersection with the DPP includes a larger online presence. In other words, the TPP benefits from significant online visibility and utilizes online campaigns, underscoring how digital communication shapes Taiwanese voter perceptions in today’s political landscape.

The NPP has a controversial image, with varying perceptions among voter groups. The second largest group (11.42%), which primarily overlaps only with the TPP, lacks a clear blue-green positioning, while other groups predominantly classify the NPP as part of the pan-green camp. In contrast, other minor parties largely remain within the traditional blue-green political spectrum, fitting into either the pan-blue or pan-green camps.

## Conclusion

This study utilized online surveys combined with MCA to cluster political parties in Taiwan and generate positioning maps that reveal nuanced party groupings. Although MCA and text mining are established techniques, their application here is distinguished by a carefully designed open-ended questionnaire that elicits voter perceptions. Traditional positioning maps typically depict parties on a two-dimensional spectrum, which provides visual clarity but may oversimplify voter views. To address this limitation, I further integrated a feature matching perspective that identifies and compares voter-perceived party characteristics, thereby enhancing the methodological framework for analyzing party similarity.

Notably, this study’s methodological approach directly addresses longstanding theoretical debates regarding the adequacy of single-dimensional models for understanding party competition in Taiwan. As established in the literature, Taiwan’s party system has been fundamentally shaped by the enduring blue-green (KMT-DPP) and unification-independence cleavages, with cross-strait relations remaining the central axis of political alignment and voter identification. While institutional factors continue to reinforce two-party dominance, the findings reveal that the emergence of new parties such as the TPP and NPP is not incidental but may reflect shifts in voter priorities. This trend highlights the need for multi-dimensional analytical frameworks that recognize both the persistent structural significance of cross-Strait relations and the meaningful impact of new party dynamics on Taiwan’s evolving political landscape.

In the Taiwanese context, this integrated approach uncovered two critical insights. First, voter perceptions in 2021 transcended traditional blue-green and unification-independence divides, with a significant proportion of respondents identifying the TPP and distinguishing the NPP from the pan-green camp. These parties are perceived as outlets for voter dissatisfaction with traditional parties, emphasizing democratic ideals alongside economic development and livelihood concerns, while also addressing localized Taiwanese identity issues amid rising social polarization and elite resentment. This signals an emerging trend toward greater diversity within Taiwan’s political landscape, although this trend still requires further observation and longitudinal validation. Second, the TPP and NPP occupy distinct positions across voter groups, introducing new axes of competition beyond traditional ideological binaries. Notably, the TPP’s pre-2022 electoral presence indicates its early establishment as a distinct political force in Taiwan’s political landscape. Meanwhile, the NPP’s image remains contentious within the pan-green camp, which may contribute to its electoral challenges.

This study has two key limitations. First, the survey, conducted in 2021, predates pivotal post-2024 election developments, including the collapsed “Blue-White Coalition” (KMT-TPP alliance), subsequent legislative collaborations, and corruption allegations against TPP leader Ko Wen-je. These developments may have reshaped public attitudes toward the TPP, which originally drew support from voters disenchanted with the two major parties. Second, while the feature matching approach provides valuable insights into Taiwan’s voter-party dynamics, its broader applicability to diverse democratic systems and party structures may be limited. Future research could extend this methodological framework to additional cases to assess its adaptability across diverse political contexts.

Overall, this research contributes to political science methodology by demonstrating how mixed-method approaches can unravel complex voter-party dynamics. It also highlights the evolving nature of Taiwan’s party system, where third forces reshape competition through non-traditional issues. Importantly, this study’s findings extend beyond the Taiwanese context by offering a replicable analytical framework for examining multi-dimensional party competition in emerging democracies. Taiwan’s experience, marked by enduring identity cleavages and the rise of populist third forces, reflects broader global trends of political fragmentation and identity-based polarization. Therefore, this research provides valuable insights for comparative politics scholars studying party system evolution, voter behavior, and the challenges of democratic consolidation in similarly divided societies. Future studies should explore how recent political realignments, such as TPP-KMT legislative collaboration, affect voter perceptions of party similarity. Further research should address the role of social media in amplifying minor parties’ visibility and reshaping electoral strategies. Finally, comparative frameworks could examine the parallels between Taiwan’s third forces and emerging parties in other East Asian democracies, highlighting broader regional trends and lessons for understanding the dynamics of political diversity.

## Supporting information

S1 Table Original survey results. (XLSX)

S2 Table The top 20 most frequent terms as criteria for political party classification. (XLSX)

S1 File Questionnaires in Chinese. This file contains the questionnaires used in the survey.
(PDF)
