# Supplementary material for: Evaluating voter perceptions of political party similarity: A mixed-method study of party positions in Taiwan
Source: PLoS One. 2025 Oct 29;20(10):e0335465. doi: 10.1371/journal.pone.0335465 (PMC12571294; doi:10.1371/journal.pone.0335465)
Supplement: S1 File — This file contains the questionnaires used in the survey. (PDF) [file pone.0335465.s003.pdf]

民眾對公民投票的看法與相關政治態度

您好！

此份網路民調是執行一項政府補助學術研究計畫案，主題為研究民眾政治參與、公民投票、公職人員選舉相關議題的看法。您的每一個回答對於研究結果而言都相當重要。當然，您個人回答的內容會完全匿名保密，僅供總體性研究參考，若有任何疑慮，您可以隨時停止作答。

希望本研究因您的貢獻而更具價值，並對於政府推行公共政策有實用幫助。預計會花費您 20 分鐘內填答完，非常感謝您的鼎力協助！

我們準備了 **Apple Watch SE 錶殼 40 公釐 GPS 版(不挑款)、小米手環 5(黑色)、必勝客六吋個人鬆厚比薩套餐即享券、統一超商 100 元電子禮券、Line Point 50 點**等獎項，將從完整填答問卷者中，公開抽出 164 個中獎名額，回饋您的用心填答，中獎名單將於 2021 年 12 月 31 日前，公布於《山水民意研究股份有限公司 Focus Survey Research》臉書專頁。謝謝您接受訪問，期待您抽到獎。

獎項	獎項內容	名額
1獎	Apple Watch SE 錶殼40公釐、GPS版、價值8,900元(不挑款)	1名
2獎	小米手環5(黑色)	3名
3獎	必勝客六吋個人鬆厚比薩套餐即享券	10名
4獎	統一超商100元電子禮券	50名
5獎	Line Point 50 點	100名
	中獎名額合計	164名

敬祝您

身體健康　心想事成

本計畫委託山水民意研究股份公司執行

【基本資料過濾題】

Q1. 請問您目前的戶籍在哪一個縣市？

(01)宜蘭縣	(02)新竹縣	(03)苗栗縣	(04)彰化縣	(05)南投縣	(06)雲林縣
(07)嘉義縣	(08)屏東縣	(09)台東縣	(10)花蓮縣	(11)澎湖縣	(12)基隆市
(13)新竹市	(14)嘉義市	(15)台北市	(16)高雄市	(17)新北市	(18)台中市
(19)台南市	(20)桃園市				

Q2. 請問您目前的戶籍在 OOO(帶入)的哪一個鄉鎮市區？

Q3. 請問您的出生年次是？(民國 93 年以後出生者中止訪問)

(網路問卷插入分頁)

【公投議題】

Q4. 您是否同意政府應全面禁止進口含有萊克多巴胺之乙型體素豬隻之肉品、內臟及其相關產製品？

(01)同意　(02)不同意

Q5. 您是否同意中油第三天然氣接收站遷離桃園大潭藻礁海岸及海域？

(01)同意　(02)不同意

Q6. 請問您對核能發電的立場是什麼？

(01)支持核四　(02)有條件支持核能發電，反對核四重啟商轉　(03)反對核能發電

(04)不知道

(網路問卷插入分頁)

【聯合分析】**(網路問卷插入分頁：Q7 ~ Q24 一題一頁)**

後續題組會針對**縣市長(粗體)**假想候選人，請您根據提供的條件挑選您比較中意的哪一位。

Q7. (縣市長 1)下列兩位假想候選人，請您根據他們的條件挑選比較中意的候選人？

「二選一，請點選按鈕」

條件	(A)	(B)
年齡	73	55
性別	男	男
本人經歷	無相關公職經歷	有擔任過民意代表
父母的政治背景	曾經在政府擔任行政要職	無
反貪腐	本身或家屬曾受到檢調貪汙調查見於媒體報導	本身或家屬曾受到檢調貪汙調查見於媒體報導
對外經貿的立場	支持美國萊豬進口，換取加強與歐美的經貿關係	支持美國萊豬進口，但強化標示與科學化管理
對同性伴侶的立場	給予同性伴侶相同的家庭權利	給予同性伴侶相同的家庭權利
市政議題上黨派合作關係	看情形藍綠皆有	泛綠
老人福利政策	注重禮金福利政策(固定節日發放小額禮金如重陽敬老金)	注重禮金福利政策(固定節日發放小額禮金如重陽敬老金)
醫療自費市場	政府應該管更多避免市場競爭	政府應該管更少交市場決定
主要青年政見	關懷勞工弱勢權益，獎勵青年創業	建設 AI 智慧城，重視青年居住問題

Q8. (縣市長 2)下列兩位假想候選人，請您根據他們的條件挑選比較中意的候選人？

Q9. (縣市長 3)下列兩位假想候選人，請您根據他們的條件挑選比較中意的候選人？

Q10. (縣市長 4)下列兩位假想候選人，請您根據他們的條件挑選比較中意的候選人？

Q11. (縣市長 5)下列兩位假想候選人，請您根據他們的條件挑選比較中意的候選人？

Q12. (縣市長 6)下列兩位假想候選人，請您根據他們的條件挑選比較中意的候選人？

後續題組會針對**縣市議員(粗體)**假想候選人，請您根據提供的條件挑選您比較中意的哪一位。

Q13. (縣市議員甲 1)下列兩位假想候選人，請您根據他們的條件挑選比較中意的候選人？

Q14. (縣市議員甲 2)下列兩位假想候選人，請您根據他們的條件挑選比較中意的候選人？

Q15. (縣市議員甲 3)下列兩位假想候選人，請您根據他們的條件挑選比較中意的候選人？

Q16. (縣市議員甲 4)下列兩位假想候選人，請您根據他們的條件挑選比較中意的候選人？

Q17. (縣市議員甲 5)下列兩位假想候選人，請您根據他們的條件挑選比較中意的候選人？

Q18. (縣市議員甲 6)下列兩位假想候選人，請您根據他們的條件挑選比較中意的候選人？

後續題組再針對**縣市議員**(粗體)假想候選人，請您根據提供的條件挑選您比較中意的哪一位。

Q19.(縣市議員乙1)下列兩位假想候選人，請您根據他們的條件挑選比較中意的候選人？

Q20.(縣市議員乙2)下列兩位假想候選人，請您根據他們的條件挑選比較中意的候選人？

Q21.(縣市議員乙3)下列兩位假想候選人，請您根據他們的條件挑選比較中意的候選人？

Q22.(縣市議員乙4)下列兩位假想候選人，請您根據他們的條件挑選比較中意的候選人？

Q23.(縣市議員乙5)下列兩位假想候選人，請您根據他們的條件挑選比較中意的候選人？

Q24.(縣市議員乙6)下列兩位假想候選人，請您根據他們的條件挑選比較中意的候選人？

(網路問卷插入分頁)

【政黨分群測試(一半樣本不納入選項10.11.12)】

Q25.許多政黨在不同議題的政策主張其實都很相像，請您把目前國內的政黨，相似性高的放在一起，當成一群。您認為和其他政黨很不一樣也可以單獨一群。如果請您用您的方式將以下國內幾個政黨分群歸類，請問您會分成幾群：_______(填入2-9) SHOW出提示政黨名稱(一半01-12；一半01-09)

(01)國民黨 (02)民進黨 (03)新黨 (04)親民黨 (05)台聯 (06)綠黨
(07)時代力量 (08)台灣基進 (09)台灣民眾黨 (10)台澎黨 (11)喜樂島聯盟
(12)統促黨

請簡單用幾個字敘述一下您的分類是根據什麼：__請說明_____(開放題)

(依分群數目列出Q26a~Q26h題目，各群已選之政黨，不會出現在下一分群的選項中)

Q26a1.以下請選出您分出之第一群，是以下幾個政黨？(選項同Q25)

Q26a2.請問您對於這群政黨(OOO、OOO帶入第一群政黨名)的分類描述是：_請說明________(開放題，非必填)

Q26b1.以下請選出您分出之第二群，是以下幾個政黨？(選項同Q25，Q26a1已選不秀出)

Q26b2.請問您對於這群政黨(OOO、OOO 帶入第二群政黨名)的分類描述是：____請說明___(開放題，非必填)

Q26c1.以下請選出您分出之第三群，是以下幾個政黨？(選項同Q25，Q26a1、Q26b1已選不秀出)

Q26c2.請問您對於這群政黨(OOO、OOO 帶入第三群政黨名)的分類描述是：_請說明_____(開放題，非必填)

Q26d1.以下請選出您分出之第四群，是以下幾個政黨？(選項同Q25，Q26a1、Q26b1、Q26c1已選不秀出)

Q26d2.請問您對於這群政黨(OOO、OOO帶入第四群政黨名)的分類描述是：___請說明___(開放題，非必填)

(網路問卷插入分頁)

Q27. 目前國內的政黨中，請問您平常在「非選舉時期」，整體而言「比較支持」(引號+粗體)哪一個政黨？

(01)國民黨 (02)民進黨 (03)新黨 (04)親民黨 (05)台聯 (06)綠黨
(07)時代力量 (08)台灣基進 (09)台灣民眾黨

Q28. 如果我們用 0 到 10 表示支持強度越來越強，請問在 0 到 10 之間，您覺得對最支持的政黨支持程度是多少：__________(填入 0-10)

Q29. 目前國內的政黨中，請問您的投票政黨傾向是下列哪一項？

(01)堅定泛藍 (02)泛藍 (03)第三勢力 (04)泛綠 (05)堅定泛綠
(06)無特定政黨傾向

Q30. 目前國內的政黨中，請問您 2020 全國立委選舉政黨票投票投給哪一個黨？

(01)國民黨 (02)民進黨 (03)新黨 (04)親民黨 (05)台聯 (06)綠黨
(07)時代力量 (08)台灣基進 (09)台灣民眾黨 (10)其他 (11)無投票/廢票/無投票權

(網路問卷插入分頁)

Q31. 目前國內的政黨中，請問以下哪些政黨是您絕對不會投票給他的？(可複選)

(01)國民黨 (02)民進黨 (03)新黨 (04)親民黨 (05)台聯 (06)綠黨
(07)時代力量 (08)台灣基進 (09)台灣民眾黨 (10)無

Q32. 如果我們用 0 到 10 表示支持強度越來越強，請問在 0 到 10 之間，您覺得對最不投的政黨支持程度是多少：__________(填入 0-10)數字必須小於/等於【Q28 最支持】

Q33. 如果我們用 0 到 10 表示支持強度越來越強，請問在 0 到 10 之間，您覺得對國民黨的政黨支持程度是多少(本題評分，請介於您「最支持 O 分(代入 Q28 答案)」與「最不投 O 分(代入 Q32 答案)」)：__________(填入 0-10)數字必須小於/等於【Q28 最支持】且大於/等於【Q32 最不投】

Q34. 如果我們用 0 到 10 表示支持強度越來越強，請問在 0 到 10 之間，您覺得對民進黨的政黨支持程度是多少(本題評分，請介於您「最支持 O 分(代入 Q28 答案)」與「最不投 O 分(代入 Q32 答案)」)：__________(填入 0-10) 數字必須小於/等於【Q28 最支持】且大於/等於【Q32 最不投】

Q34-1.不管今年中國國民黨選出的新主席是誰，您真正心目中最理想的國民黨主席是？

(01)朱立倫 (02)韓國瑜 (03)江啟臣 (04)連勝文 (05)其他，請說明______
(06)不知道 (07)不在乎

Q34-2.不管您的政黨傾向如何，請問您覺得以下哪一個人對穩定疫情最有能力？

(01)蔡英文 (02)柯文哲 (03)郭台銘 (04)侯友宜 (05)其他，請說明______
(06)不知道 (07)不在乎

(網路問卷插入分頁)

【統獨議題】

Q35. 關於臺灣和大陸的關係，有下面幾種不同的看法，請問您比較偏向那一種？

(01)儘快統一　　(02)維持現狀，以後走向統一

(03)維持現狀，看情形再決定獨立或統一

(04)永遠維持現狀

(05)維持現狀，以後走向獨立　　(06)儘快宣布獨立

(07)無意見**(網路問卷插入分頁)**

【相似性評價 A】**事後除錯機制(1)Q36-Q41 同分數；(2)相似性評價 AB 題組，對齊後相加，6 題之平均數大於 15 或小於 6 者。符合任一項即視為無效樣本。**

請問您對以下敘述的認同程度，用 0 到 10 分來表示，分數愈高代表越贊成該說法，您對以下敘述評分給多少？

Q36. 台灣民眾黨在許多政治立場上和中國國民黨相像：___________(填入 0-10)

Q37. 新黨在許多政治立場上和中國國民黨相像：___________(填入 0-10)

Q38. 台灣民眾黨在許多政治立場上和民主進步黨相像：___________(填入 0-10)

Q39. 時代力量在許多政治立場上和民主進步黨相像：___________(填入 0-10)

Q40. 台灣基進在許多政治立場上和民主進步黨相像：___________(填入 0-10)

Q41. 時代力量在許多政治立場上和中國國民黨相像：___________(填入 0-10)

(網路問卷插入分頁)

Q42. 中國國民黨在許多政治立場上和台灣民眾黨相像：___________(填入 0-10)

Q43. 民主進步黨在許多政治立場上和時代力量相像：___________(填入 0-10)

(網路問卷插入分頁)

【自我認知】**事後除錯機制：Q44-Q50 同分數 視為無效樣本。**

請問您對以下敘述的認同程度，用 0 到 10 分來表示，分數愈高代表越(贊成)認同，換句話說，您對以下敘述評分給多少？

Q44. 與一年前相比，家裡的財務狀況更糟糕或一樣不好：___________(填入 0-10)

Q45. 中國大陸出產的 COVID19 疫苗安全性堪慮，未來也不應採用：___________(填入 0-10)

Q46. 能夠成為一個政黨的黨員對個人工作或生活上是重要的：___________(填入 0-10)

Q47. 中央政府在人民納稅錢運用上有浪費的情形：___________(填入 0-10)

Q48. 中國國民黨應該改名為台灣國民黨：___________(填入 0-10)

Q49. 促進轉型正義和討黨產是重要的：___________(填入 0-10)

Q50. OOO(帶入)政府在人民納稅錢運用上有浪費的情形：___________(填入 0-10)

(網路問卷插入分頁)

Q51. 請問您在去年(2020 年)選舉期間，有沒有從事這些活動？(可複選)

(01)閱讀選舉公報
(02)閱讀候選人的傳單、快報或報刊廣告
(03)觀看候選人的電視辯論會
(04)擔任候選人或政黨的助選工作人員或義工
(05)參加候選人舉辦的活動
(06)參加候選人的後援會
(07)提醒親友觀看候選人的電視辯論會
(08)遊說或勸說別人投票給某位候選人
(09)捐款（包括購買餐券）
(10)購買候選人周邊紀念品
(11)配戴標誌或懸掛旗幟
(12)受邀參加造勢活動
(13)主動參加造勢活動
(14)瀏覽候選人網站（包含社群網站、部落格、噗浪、臉書等）　(15)都沒有

Q52. 請問您平常都從哪些管道得到新聞時事或政治資訊呢？(可複選)

(01)電視新聞台　(02)報章雜誌　(03)新聞 APP　(04)臉書朋友分享
(05)Line 朋友分享　(06)Line Today　(07)YouTube　(08)新聞網站
(09)入口網站　(10)新聞頻道臉書粉絲團　(11)親友聊天分享
(12)廣播　(13)其他-請說明　(14)都沒有

(網路問卷插入分頁)

【相似性評價 B】**事後除錯機制(1)Q53-Q58 同分數；(2)相似性評價 AB 題組，對齊後相加，6 題之平均數大於 15 或小於 6 者。符合任一項即視為無效樣本。**
請問您對以下敘述的認同程度，用 0 到 10 分來表示，分數愈高代表越贊成該說法，您對以下敘述評分給多少？

Q53. 台灣基進在許多政治立場上和民主進步黨不一樣：＿＿＿＿＿(填入 0-10)
Q54. 台灣民眾黨在許多政治立場上和民主進步黨不一樣：＿＿＿＿＿(填入 0-10)
Q55. 時代力量在許多政治立場上和民主進步黨不一樣：＿＿＿＿＿(填入 0-10)
Q56. 時代力量在許多政治立場上和中國國民黨不一樣：＿＿＿＿＿(填入 0-10)
Q57. 台灣民眾黨在許多政治立場上和中國國民黨不一樣：＿＿＿＿＿(填入 0-10)
Q58. 新黨在許多政治立場上和中國國民黨不一樣：＿＿＿＿＿(填入 0-10)

(網路問卷插入分頁)

Q59. 民主進步黨在許多政治立場上和時代力量不一樣：＿＿＿＿＿(填入 0-10)
Q60. 中國國民黨在許多政治立場上和台灣民眾黨不一樣：＿＿＿＿＿(填入 0-10)

(網路問卷插入分頁)

【政治議題自我定位】

Q61. 您在政治議題上如何定位自己的想法？

(01)非常進步　(02)有點進步　(03)中立　(04)有點保守　(05)非常保守
(06)不知道

【個人屬性測試】

Q62. 假如您可以獲得一筆意外的收入，請問您比較喜歡下列哪一種？

(01)立刻獲得３００元　　(02)一個月後獲得４５０元　　(03)不知道

Q63. 假如您可以獲得一筆意外的收入，請問您比較傾向下列哪一種？

(01)獲得 1０００元
(02)有一半的機會得２０００元，但也有一半機會得０元
(03)不知道

Q64. 假如您可能失去一筆手上的錢，請問您比較傾向下列哪一種？

(01)損失 1０００元
(02)有一半的機會損失２０００元，但也有一半機會什麼都不損失
(03)不知道

(網路問卷插入分頁)

【基本資料】

Q65. 請問您的最高學歷是什麼？

(01)小學以下 (02)國、初中 (03)高中、職 (04)專科　(05)大學　(06)碩士以上

Q66. 請問您的生理性別？

(01)男性　(02)女性

1.本研究假想縣市長候選人隨機顯示其中屬性(Level)

attribute	Levels
年齡	43， 55， 66， 73
性別	男，女
本人經歷	無相關公職經歷，有擔任過民意代表，曾有 2 任或 2 種以上公職
父母的政治背景	無、曾經擔任縣市議會議員、<br>曾經擔任中央民意代表、曾經在政府擔任行政要職
反貪腐	本身曾遭貪汙罪起訴但未判刑、本身或家屬曾受到檢調貪汙調查見於媒體報導、本人或家屬沒有涉及任何貪汙案
對外經貿的立場	支持美國萊豬進口，換取加強與歐美的經貿關係<br>支持美國萊豬進口，但強化標示與科學化管理<br>反對美國萊豬進口，應強化兩岸經貿關係<br>反對美國萊豬進口，應強化兩岸政治對話
對同性伴侶的立場	給予同性伴侶相同的家庭權利、給予同性伴侶部分的家庭權利、不給予同性伴侶任何家庭權利
市政議題上黨派合作關係	泛藍，泛綠，看情形藍綠皆有
老人福利政策	注重社工關懷政策(例如老人共餐、日間照護等)<br>注重高齡醫療政策(例如高齡免健保、免費接種疫苗等)<br>注重禮金福利政策(固定節日發放小額禮金如重陽敬老金)
醫療自費市場	政府應該管更多避免市場競爭，<br>政府應該管更少交市場決定
主要青年政見	支持政府幫你養孩子，注重下一代教育<br>建設 AI 智慧城，重視青年居住問題<br>關懷勞工弱勢權益，獎勵青年創業

2.假想議員候選人隨機顯示其中屬性(Level)

attribute	Levels

年齡	43， 55， 66， 73
性別	男，女
本人經歷	無相關公職經歷，有擔任過民意代表，曾有 2 任或 2 種以上公職
父母的政治背景	無、曾經擔任縣市議會議員、<br>曾經擔任中央民意代表、曾經在政府擔任行政要職
反貪腐	本身曾遭貪汙罪起訴但未判刑、本身或家屬曾受到檢調貪汙調查見於媒體報導、本人或家屬沒有涉及任何貪汙案
對外經貿的立場	支持美國萊豬進口，換取加強與歐美的經貿關係<br>支持美國萊豬進口，但強化標示與科學化管理<br>反對美國萊豬進口，應強化兩岸經貿關係<br>反對美國萊豬進口，應強化兩岸政治對話
對同性伴侶的立場	給予同性伴侶相同的家庭權利、給予同性伴侶部分的家庭權利、不給予同性伴侶任何家庭權利
市政議題上黨派合作關係	泛藍，泛綠，看情形藍綠皆有
醫療自費市場	政府應該管更多避免市場競爭，<br>政府應該管更少交市場決定
老人福利政策	注重社工關懷政策(例如老人共餐、日間照護等)<br>注重高齡醫療政策(例如高齡免健保、免費接種疫苗等)<br>注重禮金福利政策(固定節日發放小額禮金如重陽敬老金)
主要青年政見	支持政府幫你養孩子，注重下一代教育<br>建設 AI 智慧城，重視青年居住問題<br>關懷勞工弱勢權益，獎勵青年創業

3.假想議員候選人屬性隨機顯示其中屬性(Level)(2)

attribute	Levels
年齡	35，43， 55， 66， 73
性別	男，女
本人經歷	無相關公職經歷，有擔任過民意代表，曾有 2 任或 2 種以上公職

父母的政治背景	無、曾經擔任縣市議會議員、<br>曾經擔任中央民意代表、曾經在政府擔任行政要職
反貪腐	本身曾遭貪汙罪起訴但未判刑、本人或家屬曾受到檢調貪汙調查見於媒體報導、本人或家屬沒有涉及任何貪汙案
最高學歷	大學(含)以下，台灣碩士，留美碩士，博士
父親省籍	本省，外省籍
目前擔任職務角色	教授、企業員工、社會名流、演藝圈名人、網紅、地方政府首長、民意代表
政黨推薦	國民黨，民進黨，其他政黨，無
戶籍	在地十年以上，二年內剛遷進該選區
